# Ethyl 2,6-bis­(4-chloro­phen­yl)-4-(4-fluoro­anilino)-1-(4-fluoro­phen­yl)-1,2,5,6-tetra­hydro­pyridine-3-carboxyl­ate

**DOI:** 10.1107/S1600536813006090

**Published:** 2013-03-09

**Authors:** Sumati Anthal, Goutam Brahmachari, Suvankar Das, Rajni Kant, Vivek K. Gupta

**Affiliations:** a Post-Graduate Department of Physics & Electronics, University of Jammu, Jammu Tawi 180 006, India; b Laboratory of Natural Products & Organic Synthesis, Department of Chemistry, Visva-Bharati University, Santiniketan 731 235, West Bengal, India

## Abstract

In the title compound, C_32_H_26_Cl_2_F_2_N_2_O_2_, the tetra­hydro­pyridine ring adopts a distorted boat conformation. The chlorophenyl rings are inclined to one another by 55.2 (1)°, while for the fluorophenyl rings the dihedral angle is 80.7 (1)°. The amino group and carbonyl O atom are involved in an intra­molecular N—H⋯O hydrogen bond. In the crystal, weak C—H⋯O, C—H⋯F and C—H⋯Cl inter­actions link the mol­ecules into a three-dimensional network.

## Related literature

For the biological activity of functionalized piperidine derivatives, see: Zhou *et al.* (2007 ▶); Misra *et al.* (2009 ▶); Bin *et al.* (2001 ▶); Agrawal & Somani (2009 ▶); Dekus *et al.* (2007 ▶). For general applications of densely functionalized piperidines, see: Targum *et al.* (1995 ▶); Schotte *et al.* (1996 ▶). For general background to functionalized piperidiones, see: Desai *et al.* (1992 ▶); Pinder (1992 ▶); Watson *et al.* (2000 ▶); Breman *et al.* (2001 ▶); Kamei *et al.* (2005 ▶). For related structures, see: Sambyal *et al.* (2011 ▶); Brahmachari & Das (2012 ▶); Anthal *et al.* (2013 ▶). For asymmetry parameters, see: Duax & Norton (1975 ▶).
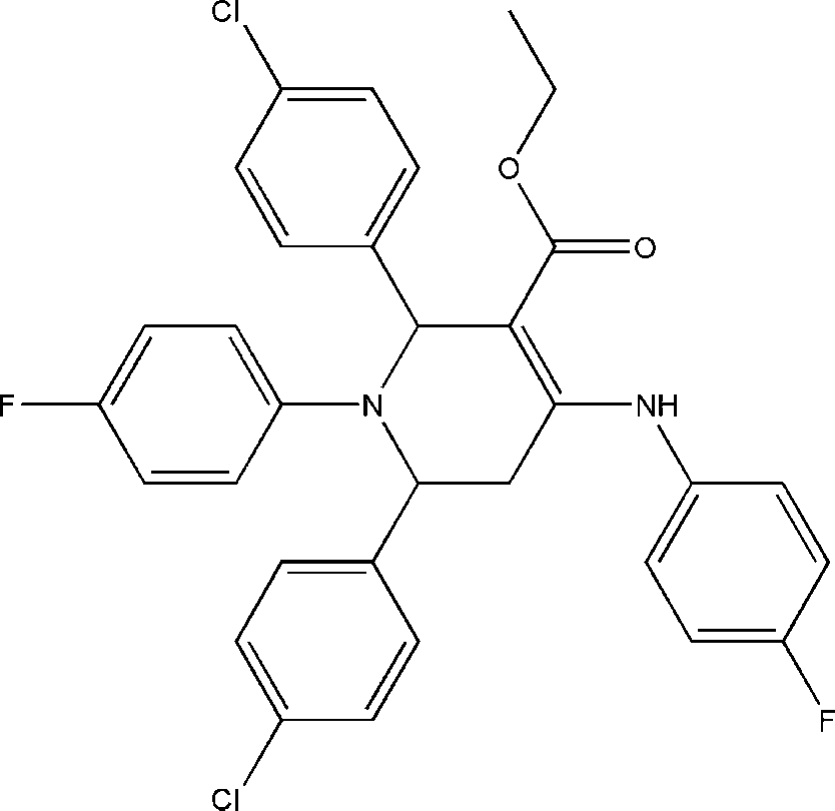


## Experimental

### 

#### Crystal data

C_32_H_26_Cl_2_F_2_N_2_O_2_
*M* *_r_* = 579.45
Triclinic, 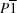

*a* = 10.3074 (7) Å
*b* = 10.7942 (5) Å
*c* = 13.9432 (10) Å
α = 103.554 (5)°
β = 106.487 (6)°
γ = 96.846 (5)°
*V* = 1417.12 (15) Å^3^
*Z* = 2
Mo *K*α radiation
μ = 0.28 mm^−1^
*T* = 293 K
0.30 × 0.20 × 0.15 mm

#### Data collection

Oxford Diffraction Xcalibur Sapphire3 diffractometer
Absorption correction: multi-scan (*CrysAlis PRO*; Oxford Diffraction, 2010 ▶) *T*_min_ = 0.881, *T*_max_ = 1.000
10718 measured reflections
5248 independent reflections
2169 reflections with *I* > 2σ(*I*)
*R*_int_ = 0.057

#### Refinement

*R*[*F* ^2^ > 2σ(*F* ^2^)] = 0.062
*wR*(*F* ^2^) = 0.133
*S* = 0.93
5248 reflections
363 parameters
H-atom parameters constrained
Δρ_max_ = 0.28 e Å^−3^
Δρ_min_ = −0.26 e Å^−3^

### 

Data collection: *CrysAlis PRO* (Oxford Diffraction, 2010 ▶); cell refinement: *CrysAlis PRO*; data reduction: *CrysAlis PRO*; program(s) used to solve structure: *SHELXS97* (Sheldrick, 2008 ▶); program(s) used to refine structure: *SHELXL97* (Sheldrick, 2008 ▶); molecular graphics: *ORTEP-3 for Windows* (Farrugia, 2012 ▶); software used to prepare material for publication: *PLATON* (Spek, 2009 ▶).

## Supplementary Material

Crystal structure: contains datablock(s) I, global. DOI: 10.1107/S1600536813006090/bg2498sup1.cif

Structure factors: contains datablock(s) I. DOI: 10.1107/S1600536813006090/bg2498Isup2.hkl

Supplementary material file. DOI: 10.1107/S1600536813006090/bg2498Isup3.cml

Additional supplementary materials:  crystallographic information; 3D view; checkCIF report

## Figures and Tables

**Table 1:** Hydrogen-bond geometry (Å, °)

*D*—H⋯*A*	*D*—H	H⋯*A*	*D*⋯*A*	*D*—H⋯*A*
N2—H2⋯O1	0.86	2.01	2.674 (4)	134
C9—H9*C*⋯Cl1^i^	0.96	2.67	3.523 (5)	148
C11—H11⋯O1^ii^	0.93	2.52	3.250 (5)	135
C18—H18⋯F1^iii^	0.93	2.52	3.259 (5)	137
C20—H20⋯F2^iv^	0.93	2.48	3.411 (4)	179

Symmetry codes: (i) 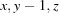; (ii) 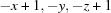; (iii) 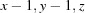; (iv) 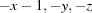.

**Fig. 1.**
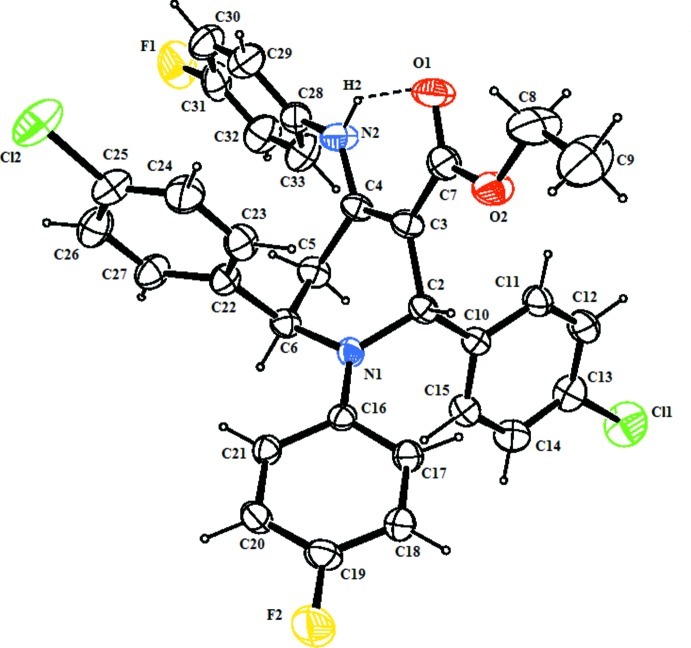
View of the molecule with the atom-labeling scheme. The thermal ellipsoids are drawn at the 40% probability level. H atoms are shown as small spheres of arbitrary radii.

**Fig. 2.**
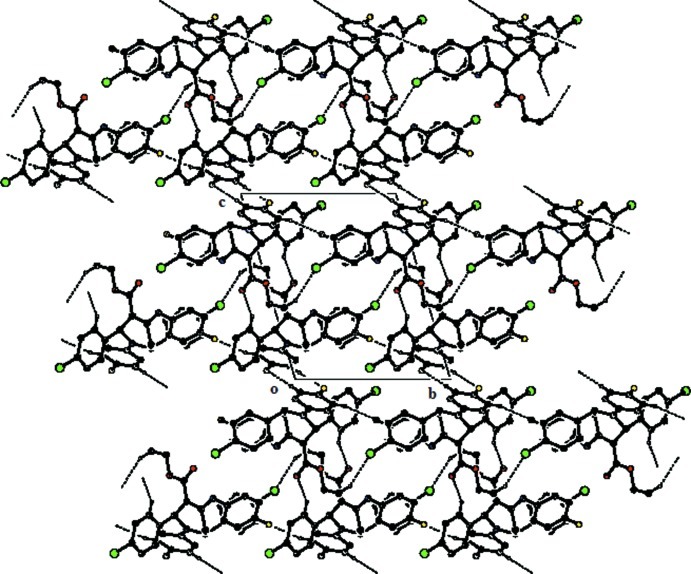
The packing arrangement of molecules viewed down the a axis.

## supplementary crystallographic information

### Comment

Functionalized piperidines are found to constitute a very important core in numerous natural products (Desai *et al.*, 1992; Pinder, 1992), synthetic pharmaceuticals (Breman *et al.*, 2001; Watson *et al.*, 2000), and a wide variety of biologically active compounds. In particular, 1,4-disubstituted piperidine scaffolds find useful applications as established drugs (Targum *et al.*, 1995; Schotte *et al.*,1996), and they exhibit a wide range of pharmacological activities including antibacterial (Zhou *et al.*, 2007), antimalarial (Misra *et al.*, 2009), anticonvulsant, anti-inflammatory (Bin *et al.*, 2001), and enzyme inhibitory activity (Agrawal & Somani, 2009; Dekus *et al.*, 2007). Moreover a large number of compounds bearing piperidine scaffold have entered into preclinical and clinical trials over the last few years (Kamei *et al.*, 2005). Hence, investigation of the structural features of biologically relevant piperidine derivatives is demanding. In continuation of our structural studies of densely functionalized piperidines (Sambyal *et al.*, 2011; Brahmachari & Das,2012; Anthal *et al.*, 2013), we present the crystal structure of ethyl 2,6-bis(4-chlorophenyl)-1-(4-fluorophenyl)-4-((4-fluorophenyl)amino)-1,2,5,6- tetrahydropyridine-3-carboxylate, determined by X-ray diffraction. In the title compound (Fig.1), the tetrahydropyridine ring adopts a distorted boat conformation with asymmetry parameters [ΔC_s_(C2)=15.9] and [ΔC_s_(C3—C4)=21.8] (Duax *et al.*, 1975). The dihedral angle between chloro-substituted phenyl rings and fluoro-substituted phenyl rings are 55.2 (1)° and 80.7 (1)°. An intramolecular hydrogen bond N2—H2···O1 is found. This interaction leads to the formation of a pseudo-six membered ring comprising atoms O1, C7, C3, C4, N2 and H2.Weak intermolecular C—H···O, C—H···F and C—H···Cl interactions join molecules into a three-dimensional network (Table 1). A packing view down the *a* axis is shown in Fig.2.

### Experimental

An oven-dried screw cap reaction tube was charged with a magnetic stir bar, 4-fluoroaniline (2 mmol), ethyl acetoacetate (1 mmol) and Bi(NO_3_)_3_.5H_2_O (10 mol%) in 4 ml e thanol; the mixture was stirred at room temperature for 20 min, and afterthen 4-chlorobenzaldehyde (2 mmol) was added to the reaction mixture and stirring was continued up to 18 h to complete the reaction (monitored by TLC). On completion of the reaction, a thick white precipitate was obtained. The solid residue was filtered off and washed with cold ethanol-water. The solid mass was dissolved in hot ethyl acetate-ethanol mixture and filtered off when bismuth salt separated out; the filtrate on standing afforded white crystals of the title compound, characterized by elemental analyses and spectral studies including FT—IR, ^1^H-NMR, and ^13^C-NMR. For X-ray study, white single crystals of title compound (mp 219–222 °C) were prepared by further recrystallization by slow evaporation from ethanol-ethyl acetate-water solution.

### Refinement

All H atoms were positioned geometrically and were treated as riding on their parent C/N atoms, with C—H distances of 0.93–0.98 Å and N—H distance of 0.86 Å; and with *U*_iso_(H) = 1.2*U*_eq_(C/N), except for the methyl groups where *U*_iso_(H) = 1.5*U*_eq_(C),. The poor diffraction quality of the crystals prevented the obtention of a better data set with a larger N_obs_/N_total_ reflection ratio.

### Crystal data

**Table:**

C_32_H_26_Cl_2_F_2_N_2_O_2_	*Z* = 2
*M**_r_* = 579.45	*F*(000) = 600
Triclinic, *P*1	*D*_x_ = 1.358 Mg m^−^^3^
Hall symbol: -P 1	Mo *K*α radiation, λ = 0.71073 Å
*a* = 10.3074 (7) Å	Cell parameters from 3771 reflections
*b* = 10.7942 (5) Å	θ = 3.5–29.0°
*c* = 13.9432 (10) Å	µ = 0.28 mm^−^^1^
α = 103.554 (5)°	*T* = 293 K
β = 106.487 (6)°	Plate, white
γ = 96.846 (5)°	0.30 × 0.20 × 0.15 mm
*V* = 1417.12 (15) Å^3^	

### Data collection

**Table:**

Oxford Diffraction Xcalibur Sapphire3 diffractometer	5248 independent reflections
Radiation source: fine-focus sealed tube	2169 reflections with *I* > 2σ(*I*)
Graphite monochromator	*R*_int_ = 0.057
Detector resolution: 16.1049 pixels mm^-1^	θ_max_ = 25.5°, θ_min_ = 3.5°
ω scans	*h* = −12→12
Absorption correction: multi-scan (*CrysAlis PRO*; Oxford Diffraction, 2010)	*k* = −13→12
*T*_min_ = 0.881, *T*_max_ = 1.000	*l* = −16→16
10718 measured reflections	

### Refinement

**Table:**

Refinement on *F*^2^	Secondary atom site location: difference Fourier map
Least-squares matrix: full	Hydrogen site location: inferred from neighbouring sites
*R*[*F*^2^ > 2σ(*F*^2^)] = 0.062	H-atom parameters constrained
*wR*(*F*^2^) = 0.133	*w* = 1/[σ^2^(*F*_o_^2^) + (0.029*P*)^2^] where *P* = (*F*_o_^2^ + 2*F*_c_^2^)/3
*S* = 0.93	(Δ/σ)_max_ = 0.001
5248 reflections	Δρ_max_ = 0.28 e Å^−^^3^
363 parameters	Δρ_min_ = −0.26 e Å^−^^3^
0 restraints	Extinction correction: *SHELXL97* (Sheldrick, 2008), Fc^*^=kFc[1+0.001xFc^2^λ^3^/sin(2θ)]^-1/4^
Primary atom site location: structure-invariant direct methods	Extinction coefficient: 0.0046 (8)

### Special details

**Table:**

Experimental. *CrysAlis PRO*, Oxford Diffraction Ltd., Version 1.171.34.40 (release 27–08-2010 CrysAlis171. NET) (compiled Aug 27 2010,11:50:40) Empirical absorption correction using spherical harmonics, implemented in SCALE3 ABSPACK scaling algorithm.
Geometry. All e.s.d.'s (except the e.s.d. in the dihedral angle between two l.s. planes) are estimated using the full covariance matrix. The cell e.s.d.'s are taken into account individually in the estimation of e.s.d.'s in distances, angles and torsion angles; correlations between e.s.d.'s in cell parameters are only used when they are defined by crystal symmetry. An approximate (isotropic) treatment of cell e.s.d.'s is used for estimating e.s.d.'s involving l.s. planes.
Refinement. Refinement of *F*^2^ against ALL reflections. The weighted *R*-factor *wR* and goodness of fit *S* are based on *F*^2^, conventional *R*-factors *R* are based on *F*, with *F* set to zero for negative *F*^2^. The threshold expression of *F*^2^ > σ(*F*^2^) is used only for calculating *R*-factors(gt) *etc*. and is not relevant to the choice of reflections for refinement. *R*-factors based on *F*^2^ are statistically about twice as large as those based on *F*, and *R*- factors based on ALL data will be even larger.

### Fractional atomic coordinates and isotropic or equivalent isotropic displacement parameters (Å^2^)

**Table:**

	*x*	*y*	*z*	*U*_iso_*/*U*_eq_	
Cl1	0.08233 (15)	0.65481 (11)	0.39945 (11)	0.1144 (6)	
Cl2	0.37360 (13)	−0.49679 (12)	0.06250 (10)	0.0983 (5)	
F1	0.7899 (3)	0.5548 (3)	0.2179 (2)	0.1196 (11)	
F2	−0.4881 (2)	−0.1674 (2)	0.0483 (2)	0.0936 (9)	
O1	0.4233 (3)	0.1728 (3)	0.5195 (2)	0.0778 (9)	
O2	0.2475 (3)	0.0078 (3)	0.4821 (2)	0.0726 (9)	
N1	0.0762 (3)	0.0160 (2)	0.1940 (2)	0.0424 (7)	
N2	0.4685 (3)	0.2445 (3)	0.3589 (2)	0.0608 (9)	
H2	0.4906	0.2599	0.4255	0.073*	
C2	0.1699 (3)	−0.0372 (3)	0.2673 (2)	0.0421 (9)	
H2A	0.1179	−0.0684	0.3088	0.051*	
C3	0.2907 (4)	0.0690 (3)	0.3426 (3)	0.0466 (10)	
C4	0.3580 (4)	0.1479 (3)	0.3018 (3)	0.0436 (9)	
C5	0.2907 (4)	0.1349 (3)	0.1887 (3)	0.0517 (10)	
H5A	0.3069	0.0570	0.1464	0.062*	
H5B	0.3297	0.2092	0.1708	0.062*	
C6	0.1341 (4)	0.1273 (3)	0.1672 (3)	0.0451 (10)	
H6	0.0910	0.1136	0.0922	0.054*	
C7	0.3280 (4)	0.0887 (4)	0.4538 (3)	0.0576 (11)	
C8	0.2793 (6)	0.0239 (5)	0.5942 (3)	0.117 (2)	
H8A	0.3724	0.0107	0.6229	0.141*	
H8B	0.2752	0.1117	0.6288	0.141*	
C9	0.1875 (6)	−0.0635 (5)	0.6127 (4)	0.133 (2)	
H9A	0.0965	−0.0453	0.5901	0.200*	
H9B	0.2143	−0.0559	0.6860	0.200*	
H9C	0.1874	−0.1502	0.5750	0.200*	
C10	0.2196 (3)	−0.1527 (3)	0.2129 (3)	0.0431 (9)	
C11	0.3258 (4)	−0.1993 (3)	0.2690 (3)	0.0543 (11)	
H11	0.3661	−0.1589	0.3400	0.065*	
C12	0.3748 (4)	−0.3026 (4)	0.2244 (3)	0.0618 (12)	
H12	0.4473	−0.3311	0.2645	0.074*	
C13	0.3155 (4)	−0.3634 (3)	0.1200 (3)	0.0593 (11)	
C14	0.2075 (4)	−0.3228 (4)	0.0607 (3)	0.0593 (11)	
H14	0.1665	−0.3651	−0.0099	0.071*	
C15	0.1605 (4)	−0.2175 (3)	0.1079 (3)	0.0501 (10)	
H15	0.0873	−0.1896	0.0679	0.060*	
C16	−0.0649 (4)	−0.0309 (3)	0.1574 (2)	0.0378 (9)	
C17	−0.1231 (4)	−0.1478 (3)	0.1688 (3)	0.0465 (10)	
H17	−0.0655	−0.1967	0.2009	0.056*	
C18	−0.2644 (4)	−0.1928 (3)	0.1334 (3)	0.0536 (10)	
H18	−0.3017	−0.2696	0.1436	0.064*	
C19	−0.3481 (4)	−0.1232 (4)	0.0837 (3)	0.0565 (11)	
C20	−0.2965 (4)	−0.0084 (4)	0.0697 (3)	0.0510 (10)	
H20	−0.3557	0.0384	0.0365	0.061*	
C21	−0.1567 (4)	0.0368 (3)	0.1052 (2)	0.0414 (9)	
H21	−0.1215	0.1140	0.0945	0.050*	
C22	0.1100 (4)	0.2574 (3)	0.2241 (3)	0.0455 (9)	
C23	0.0850 (4)	0.2775 (4)	0.3179 (3)	0.0568 (11)	
H23	0.0746	0.2077	0.3453	0.068*	
C24	0.0751 (4)	0.3986 (4)	0.3724 (3)	0.0686 (13)	
H24	0.0604	0.4109	0.4365	0.082*	
C25	0.0871 (4)	0.4993 (4)	0.3305 (4)	0.0681 (13)	
C26	0.1069 (4)	0.4825 (4)	0.2349 (4)	0.0714 (13)	
H26	0.1126	0.5518	0.2062	0.086*	
C27	0.1183 (4)	0.3608 (3)	0.1825 (3)	0.0593 (11)	
H27	0.1317	0.3484	0.1180	0.071*	
C28	0.5515 (4)	0.3228 (4)	0.3226 (3)	0.0526 (10)	
C29	0.5940 (4)	0.4541 (4)	0.3719 (3)	0.0732 (13)	
H29	0.5665	0.4909	0.4283	0.088*	
C30	0.6777 (5)	0.5317 (4)	0.3375 (4)	0.0826 (16)	
H30	0.7090	0.6200	0.3715	0.099*	
C31	0.7127 (5)	0.4760 (5)	0.2533 (5)	0.0763 (14)	
C32	0.6739 (4)	0.3486 (5)	0.2043 (4)	0.0796 (14)	
H32	0.7008	0.3133	0.1472	0.095*	
C33	0.5942 (4)	0.2713 (4)	0.2393 (3)	0.0679 (12)	
H33	0.5683	0.1824	0.2064	0.082*	

### Atomic displacement parameters (Å^2^)

**Table:**

	*U*^11^	*U*^22^	*U*^33^	*U*^12^	*U*^13^	*U*^23^
Cl1	0.1493 (13)	0.0584 (8)	0.1198 (12)	0.0113 (8)	0.0527 (10)	−0.0108 (7)
Cl2	0.1033 (10)	0.0923 (9)	0.1124 (10)	0.0470 (8)	0.0568 (8)	0.0128 (7)
F1	0.094 (2)	0.123 (2)	0.158 (3)	−0.0029 (18)	0.0331 (19)	0.092 (2)
F2	0.0473 (16)	0.0999 (19)	0.126 (2)	−0.0009 (14)	0.0046 (15)	0.0539 (16)
O1	0.080 (2)	0.082 (2)	0.0453 (17)	−0.0184 (17)	0.0000 (16)	0.0110 (15)
O2	0.094 (2)	0.0760 (19)	0.0384 (16)	−0.0104 (17)	0.0182 (16)	0.0153 (14)
N1	0.0394 (19)	0.0418 (17)	0.0472 (19)	0.0047 (15)	0.0096 (15)	0.0216 (14)
N2	0.059 (2)	0.068 (2)	0.0428 (19)	−0.0132 (19)	0.0114 (17)	0.0100 (16)
C2	0.041 (2)	0.050 (2)	0.038 (2)	0.0097 (18)	0.0127 (17)	0.0156 (16)
C3	0.048 (2)	0.056 (2)	0.034 (2)	0.008 (2)	0.0128 (18)	0.0104 (17)
C4	0.042 (2)	0.056 (2)	0.033 (2)	0.0074 (19)	0.0128 (18)	0.0122 (17)
C5	0.054 (3)	0.060 (2)	0.045 (2)	0.010 (2)	0.0209 (19)	0.0164 (18)
C6	0.048 (2)	0.051 (2)	0.036 (2)	0.0056 (19)	0.0142 (18)	0.0134 (17)
C7	0.066 (3)	0.061 (3)	0.049 (3)	0.010 (2)	0.019 (2)	0.019 (2)
C8	0.174 (6)	0.116 (4)	0.035 (3)	−0.050 (4)	0.028 (3)	0.017 (3)
C9	0.223 (7)	0.105 (4)	0.069 (4)	−0.015 (4)	0.070 (4)	0.014 (3)
C10	0.039 (2)	0.047 (2)	0.041 (2)	0.0086 (18)	0.0096 (18)	0.0126 (17)
C11	0.055 (3)	0.051 (2)	0.051 (2)	0.021 (2)	0.008 (2)	0.0102 (19)
C12	0.047 (3)	0.065 (3)	0.071 (3)	0.022 (2)	0.010 (2)	0.020 (2)
C13	0.060 (3)	0.059 (3)	0.072 (3)	0.023 (2)	0.038 (2)	0.015 (2)
C14	0.061 (3)	0.069 (3)	0.047 (2)	0.012 (2)	0.024 (2)	0.007 (2)
C15	0.047 (2)	0.058 (2)	0.050 (2)	0.014 (2)	0.0183 (19)	0.0178 (19)
C16	0.045 (2)	0.0358 (19)	0.0293 (19)	0.0044 (18)	0.0107 (17)	0.0060 (15)
C17	0.043 (2)	0.043 (2)	0.051 (2)	0.0158 (19)	0.0120 (19)	0.0102 (17)
C18	0.058 (3)	0.048 (2)	0.053 (2)	0.004 (2)	0.012 (2)	0.0206 (18)
C19	0.038 (3)	0.066 (3)	0.056 (3)	0.003 (2)	0.005 (2)	0.015 (2)
C20	0.047 (3)	0.059 (3)	0.046 (2)	0.014 (2)	0.007 (2)	0.0209 (19)
C21	0.045 (2)	0.039 (2)	0.041 (2)	0.0075 (18)	0.0148 (18)	0.0139 (16)
C22	0.048 (2)	0.044 (2)	0.048 (2)	0.0048 (18)	0.0212 (19)	0.0164 (18)
C23	0.064 (3)	0.059 (3)	0.055 (3)	0.010 (2)	0.024 (2)	0.024 (2)
C24	0.092 (4)	0.066 (3)	0.049 (3)	0.019 (3)	0.028 (2)	0.010 (2)
C25	0.077 (3)	0.048 (3)	0.071 (3)	0.003 (2)	0.025 (3)	0.003 (2)
C26	0.076 (3)	0.052 (3)	0.092 (4)	0.003 (2)	0.035 (3)	0.026 (2)
C27	0.071 (3)	0.048 (2)	0.061 (3)	0.001 (2)	0.029 (2)	0.016 (2)
C28	0.043 (2)	0.054 (3)	0.061 (3)	0.007 (2)	0.015 (2)	0.019 (2)
C29	0.068 (3)	0.059 (3)	0.084 (3)	0.008 (3)	0.024 (3)	0.007 (2)
C30	0.074 (4)	0.052 (3)	0.104 (4)	−0.004 (3)	0.014 (3)	0.017 (3)
C31	0.058 (3)	0.075 (4)	0.108 (4)	0.005 (3)	0.022 (3)	0.058 (3)
C32	0.068 (3)	0.075 (3)	0.101 (4)	0.005 (3)	0.038 (3)	0.026 (3)
C33	0.065 (3)	0.057 (3)	0.092 (4)	0.004 (2)	0.043 (3)	0.022 (2)

### Geometric parameters (Å, º)

**Table:**

Cl1—C25	1.742 (4)	C12—H12	0.9300
Cl2—C13	1.735 (3)	C13—C14	1.371 (5)
F1—C31	1.364 (5)	C14—C15	1.386 (4)
F2—C19	1.369 (4)	C14—H14	0.9300
O1—C7	1.230 (4)	C15—H15	0.9300
O2—C7	1.335 (5)	C16—C17	1.395 (4)
O2—C8	1.466 (5)	C16—C21	1.407 (4)
N1—C16	1.384 (4)	C17—C18	1.383 (4)
N1—C6	1.450 (4)	C17—H17	0.9300
N1—C2	1.468 (4)	C18—C19	1.359 (4)
N2—C4	1.353 (4)	C18—H18	0.9300
N2—C28	1.406 (5)	C19—C20	1.368 (5)
N2—H2	0.8600	C20—C21	1.369 (4)
C2—C3	1.523 (4)	C20—H20	0.9300
C2—C10	1.524 (4)	C21—H21	0.9300
C2—H2A	0.9800	C22—C23	1.377 (5)
C3—C4	1.356 (5)	C22—C27	1.377 (5)
C3—C7	1.444 (5)	C23—C24	1.381 (4)
C4—C5	1.497 (4)	C23—H23	0.9300
C5—C6	1.545 (4)	C24—C25	1.358 (5)
C5—H5A	0.9700	C24—H24	0.9300
C5—H5B	0.9700	C25—C26	1.379 (5)
C6—C22	1.529 (4)	C26—C27	1.382 (4)
C6—H6	0.9800	C26—H26	0.9300
C8—C9	1.376 (6)	C27—H27	0.9300
C8—H8A	0.9700	C28—C29	1.377 (5)
C8—H8B	0.9700	C28—C33	1.378 (5)
C9—H9A	0.9600	C29—C30	1.388 (6)
C9—H9B	0.9600	C29—H29	0.9300
C9—H9C	0.9600	C30—C31	1.353 (6)
C10—C11	1.376 (4)	C30—H30	0.9300
C10—C15	1.385 (4)	C31—C32	1.338 (5)
C11—C12	1.368 (4)	C32—C33	1.366 (5)
C11—H11	0.9300	C32—H32	0.9300
C12—C13	1.369 (5)	C33—H33	0.9300
			
C7—O2—C8	116.7 (3)	C13—C14—H14	120.6
C16—N1—C6	120.2 (3)	C15—C14—H14	120.6
C16—N1—C2	121.6 (3)	C10—C15—C14	121.7 (3)
C6—N1—C2	118.1 (3)	C10—C15—H15	119.1
C4—N2—C28	127.9 (3)	C14—C15—H15	119.1
C4—N2—H2	116.0	N1—C16—C17	121.9 (3)
C28—N2—H2	116.0	N1—C16—C21	121.3 (3)
N1—C2—C3	110.7 (3)	C17—C16—C21	116.8 (3)
N1—C2—C10	112.9 (3)	C18—C17—C16	121.5 (3)
C3—C2—C10	111.4 (3)	C18—C17—H17	119.3
N1—C2—H2A	107.2	C16—C17—H17	119.3
C3—C2—H2A	107.2	C19—C18—C17	119.1 (4)
C10—C2—H2A	107.2	C19—C18—H18	120.5
C4—C3—C7	121.2 (3)	C17—C18—H18	120.5
C4—C3—C2	117.7 (3)	C18—C19—C20	121.8 (4)
C7—C3—C2	121.1 (4)	C18—C19—F2	119.4 (4)
N2—C4—C3	124.2 (3)	C20—C19—F2	118.8 (3)
N2—C4—C5	119.8 (3)	C19—C20—C21	119.2 (3)
C3—C4—C5	115.5 (3)	C19—C20—H20	120.4
C4—C5—C6	108.9 (3)	C21—C20—H20	120.4
C4—C5—H5A	109.9	C20—C21—C16	121.6 (3)
C6—C5—H5A	109.9	C20—C21—H21	119.2
C4—C5—H5B	109.9	C16—C21—H21	119.2
C6—C5—H5B	109.9	C23—C22—C27	118.1 (3)
H5A—C5—H5B	108.3	C23—C22—C6	122.6 (3)
N1—C6—C22	114.4 (3)	C27—C22—C6	119.2 (3)
N1—C6—C5	109.7 (3)	C22—C23—C24	121.8 (4)
C22—C6—C5	109.1 (3)	C22—C23—H23	119.1
N1—C6—H6	107.8	C24—C23—H23	119.1
C22—C6—H6	107.8	C25—C24—C23	118.7 (4)
C5—C6—H6	107.8	C25—C24—H24	120.7
O1—C7—O2	121.0 (4)	C23—C24—H24	120.7
O1—C7—C3	124.9 (4)	C24—C25—C26	121.5 (4)
O2—C7—C3	114.1 (4)	C24—C25—Cl1	120.0 (4)
C9—C8—O2	110.8 (4)	C26—C25—Cl1	118.5 (4)
C9—C8—H8A	109.5	C25—C26—C27	118.7 (4)
O2—C8—H8A	109.5	C25—C26—H26	120.7
C9—C8—H8B	109.5	C27—C26—H26	120.7
O2—C8—H8B	109.5	C22—C27—C26	121.2 (4)
H8A—C8—H8B	108.1	C22—C27—H27	119.4
C8—C9—H9A	109.5	C26—C27—H27	119.4
C8—C9—H9B	109.5	C29—C28—C33	118.5 (4)
H9A—C9—H9B	109.5	C29—C28—N2	119.9 (4)
C8—C9—H9C	109.5	C33—C28—N2	121.6 (4)
H9A—C9—H9C	109.5	C28—C29—C30	120.2 (4)
H9B—C9—H9C	109.5	C28—C29—H29	119.9
C11—C10—C15	116.8 (3)	C30—C29—H29	119.9
C11—C10—C2	120.1 (3)	C31—C30—C29	118.5 (4)
C15—C10—C2	123.1 (3)	C31—C30—H30	120.8
C12—C11—C10	122.7 (3)	C29—C30—H30	120.8
C12—C11—H11	118.7	C32—C31—C30	122.7 (5)
C10—C11—H11	118.7	C32—C31—F1	119.7 (5)
C11—C12—C13	119.1 (3)	C30—C31—F1	117.6 (5)
C11—C12—H12	120.4	C31—C32—C33	119.1 (5)
C13—C12—H12	120.4	C31—C32—H32	120.5
C12—C13—C14	120.7 (3)	C33—C32—H32	120.5
C12—C13—Cl2	119.9 (3)	C32—C33—C28	121.0 (4)
C14—C13—Cl2	119.4 (3)	C32—C33—H33	119.5
C13—C14—C15	118.9 (3)	C28—C33—H33	119.5
			
C16—N1—C2—C3	−145.8 (3)	C13—C14—C15—C10	0.0 (6)
C6—N1—C2—C3	29.1 (4)	C6—N1—C16—C17	170.8 (3)
C16—N1—C2—C10	88.6 (4)	C2—N1—C16—C17	−14.4 (5)
C6—N1—C2—C10	−96.4 (3)	C6—N1—C16—C21	−8.2 (5)
N1—C2—C3—C4	−48.0 (4)	C2—N1—C16—C21	166.6 (3)
C10—C2—C3—C4	78.4 (4)	N1—C16—C17—C18	178.9 (3)
N1—C2—C3—C7	129.1 (3)	C21—C16—C17—C18	−2.1 (5)
C10—C2—C3—C7	−104.5 (4)	C16—C17—C18—C19	2.0 (5)
C28—N2—C4—C3	173.8 (3)	C17—C18—C19—C20	−1.6 (6)
C28—N2—C4—C5	−14.8 (5)	C17—C18—C19—F2	179.9 (3)
C7—C3—C4—N2	4.2 (5)	C18—C19—C20—C21	1.1 (6)
C2—C3—C4—N2	−178.7 (3)	F2—C19—C20—C21	179.7 (3)
C7—C3—C4—C5	−167.5 (3)	C19—C20—C21—C16	−1.2 (5)
C2—C3—C4—C5	9.6 (4)	N1—C16—C21—C20	−179.3 (3)
N2—C4—C5—C6	−128.3 (3)	C17—C16—C21—C20	1.7 (5)
C3—C4—C5—C6	43.7 (4)	N1—C6—C22—C23	23.6 (5)
C16—N1—C6—C22	73.7 (4)	C5—C6—C22—C23	−99.8 (4)
C2—N1—C6—C22	−101.3 (3)	N1—C6—C22—C27	−158.9 (3)
C16—N1—C6—C5	−163.3 (3)	C5—C6—C22—C27	77.8 (4)
C2—N1—C6—C5	21.7 (4)	C27—C22—C23—C24	−3.2 (6)
C4—C5—C6—N1	−59.6 (4)	C6—C22—C23—C24	174.4 (3)
C4—C5—C6—C22	66.5 (4)	C22—C23—C24—C25	1.6 (6)
C8—O2—C7—O1	−0.5 (6)	C23—C24—C25—C26	0.9 (7)
C8—O2—C7—C3	−179.5 (4)	C23—C24—C25—Cl1	−177.3 (3)
C4—C3—C7—O1	−2.5 (6)	C24—C25—C26—C27	−1.7 (7)
C2—C3—C7—O1	−179.5 (3)	Cl1—C25—C26—C27	176.6 (3)
C4—C3—C7—O2	176.5 (3)	C23—C22—C27—C26	2.3 (6)
C2—C3—C7—O2	−0.5 (5)	C6—C22—C27—C26	−175.4 (3)
C7—O2—C8—C9	178.9 (4)	C25—C26—C27—C22	0.1 (6)
N1—C2—C10—C11	168.6 (3)	C4—N2—C28—C29	138.4 (4)
C3—C2—C10—C11	43.4 (5)	C4—N2—C28—C33	−42.7 (6)
N1—C2—C10—C15	−12.9 (5)	C33—C28—C29—C30	0.1 (6)
C3—C2—C10—C15	−138.2 (4)	N2—C28—C29—C30	179.0 (4)
C15—C10—C11—C12	1.4 (6)	C28—C29—C30—C31	1.9 (7)
C2—C10—C11—C12	180.0 (4)	C29—C30—C31—C32	−2.4 (7)
C10—C11—C12—C13	−0.5 (6)	C29—C30—C31—F1	177.2 (4)
C11—C12—C13—C14	−0.7 (6)	C30—C31—C32—C33	0.9 (7)
C11—C12—C13—Cl2	−178.7 (3)	F1—C31—C32—C33	−178.7 (4)
C12—C13—C14—C15	1.0 (6)	C31—C32—C33—C28	1.2 (7)
Cl2—C13—C14—C15	179.0 (3)	C29—C28—C33—C32	−1.7 (6)
C11—C10—C15—C14	−1.1 (6)	N2—C28—C33—C32	179.4 (4)
C2—C10—C15—C14	−179.6 (3)		

### Hydrogen-bond geometry (Å, º)

**Table:**

*D*—H···*A*	*D*—H	H···*A*	*D*···*A*	*D*—H···*A*
N2—H2···O1	0.86	2.01	2.674 (4)	134
C9—H9*C*···Cl1^i^	0.96	2.67	3.523 (5)	148
C11—H11···O1^ii^	0.93	2.52	3.250 (5)	135
C18—H18···F1^iii^	0.93	2.52	3.259 (5)	137
C20—H20···F2^iv^	0.93	2.48	3.411 (4)	179

Symmetry codes: (i) *x*, *y*−1, *z*; (ii) −*x*+1, −*y*, −*z*+1; (iii) *x*−1, *y*−1, *z*; (iv) −*x*−1, −*y*, −*z*.
